# Inactivated Sendai Virus (HVJ-E) Immobilized Electrospun Nanofiber for Cancer Therapy

**DOI:** 10.3390/ma9010012

**Published:** 2015-12-26

**Authors:** Takaharu Okada, Eri Niiyama, Koichiro Uto, Takao Aoyagi, Mitsuhiro Ebara

**Affiliations:** 1 Graduate School of Pure and Applied Sciences, University of Tsukuba, 1-1-1, Tennodai, Tsukuba, Ibaraki 305-8577, Japan; OKADA.Takaharu@nims.go.jp (T.O.); NIYAMA.Eri@nims.go.jp (E.N.); 2 Biomaterials Unit, Nano-Life Field, International Center for Materials Nanoarchitectonics (WPI-MANA), National Institute for Materials Science (NIMS), 1-1 Namiki, Tsukuba, Ibaraki 305-0044, Japan; flatronl1753s@gmail.com (K.U.); aoyagi.takao@nihon-u.ac.jp (T.A.); 3 Japan Society for the Promotion of Science (JSPS), 8 Ichibancho, Chiyoda-ku, Tokyo 102-0083, Japan; 4 Graduate School of Tokyo University of Science, 6-3-1 Niijuku, Katsushika-ku, Tokyo 125-8585, Japan

**Keywords:** nanofiber, layer-by-layer, inactivated Sendai virus, HVJ-E

## Abstract

Inactivated Hemagglutinating Virus of Japan Envelope (HVJ-E) was immobilized on electrospun nanofibers of poly(*ε*-caprolactone) by layer-by-layer (LbL) assembly technique. The precursor LbL film was first constructed with poly-L-lysine and alginic acid via electrostatic interaction. Then the HVJ-E particles were immobilized on the cationic PLL outermost surface. The HVJ-E adsorption was confirmed by surface wettability test, scanning laser microscopy, scanning electron microscopy, and confocal laser microscopy. The immobilized HVJ-E particles were released from the nanofibers under physiological condition. *In vitro* cytotoxic assay demonstrated that the released HVJ-E from nanofibers induced cancer cell deaths. This surface immobilization technique is possible to perform on anti-cancer drug incorporated nanofibers that enables the fibers to show chemotherapy and immunotherapy simultaneously for an effective eradication of tumor cells *in vivo*.

## 1. Introduction

According to the world healthcare organization’s (WHO) report in 2012, 8.2 million deaths worldwide could be attributed to cancer [1]. Of these, nearly 45% of new cases were diagnosed in Asia. In Japan, cancer has been the top cause of death since 1981. Strategies to decrease the number of deaths caused by cancer, therefore, remain a high priority in medical research. The cancer treatment options depend on the type and stage of cancer, possible side effects, and the patient's preferences and overall health. There are many cancer treatment methods that have been developed such as surgery, radiotherapy, and chemotherapy. Depending on the cancer type and location, severances, and potential to metastasize, medical doctors will choose one or a combination of the treatments. For example, systematic chemotherapy along with surgery or radiotherapy are the most commonly used therapeutic strategies for cancer [2]. Hyperthermia has also been combined with chemotherapy or radiotherapy, because the application of hyperthermia has been known to cause manifold cytotoxic effects to tumor cells [3,4]. We have recently developed a unique nanofiber mesh that can run both chemotherapy and hyperthermia treatment at the same time [5]. The fibers are composed of a temperature responsive *N*-isopropylacrylamide (NIPAAm) [6,7] based copolymer, anti-cancer drugs, and magnetic nanoparticles (MNPs). When an alternative magnetic field is applied to the fibers, the fibers shrink because of self-generated heat from the incorporated MNPs this induces the deswelling of polymer networks in the nanofiber [5,8]. The contraction of the fibers expels the incorporated anti-cancer drugs directly into the cancer area. Applying an alternative magnetic field induces both direct heating of cancer cells and the subsequent release of anti-cancer drugs. The synergistic effect of chemotherapy and hyperthermia treatment showed virtual eradication of the tumor cells *in vitro* compared with individual treatment. 

**Figure 1:**
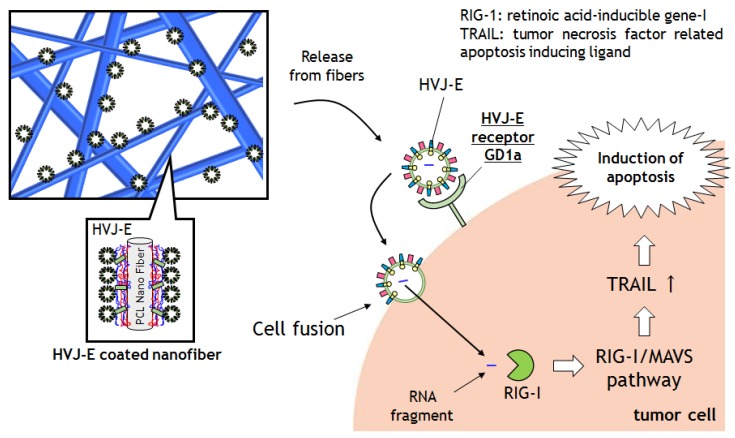
Schematic illustrations of HVJ-E coated nanofiber meshes and the mechanism of cancer apoptosis induced by the released HVJ-E particles from nanofiber.

In recent years, cancer immunotherapy has emerged as the fourth treatment modality, in addition to surgery, chemotherapy, and radiotherapy [9,10]. The main types of immunotherapy now being used include monoclonal antibodies, immune checkpoint inhibitors, cancer vaccines, or other non-specific immunotherapies. Kaneda *et al*. have recently shown a new type of immune treatment using inactivated Hemagglutinating Virus of Japan Envelope (HVJ-E). HVJ-E is prepared by irradiating UV light to destroy the internal RNA of the HVJ (or Sendai) virus [11]. Although the virus is no longer cytotoxic and is unable to proliferate, however, the cell fusion functionality remains active. Recently, this fusion ability has been demonstrated in the literature for the delivery of artificially encapsulated plasmids or proteins to cells [12,13,14]. It has been also reported that HVJ-E can induce tumor specific apoptosis and anti-cancer immunity *in vitro* and *in vivo*. Kurooka *et al*. reported an injected HVJ-E particle induces interleukin-6 (IL-6) production that enhances a strong anti-tumor immunity *in vivo* [15,16]. The HVJ-E is also able to induce cell selective tumor apoptosis *in vitro* which was reported by Kawaguchi *et al*. [17]. They seeded HVJ-E on prostatic tumor cells, PC-3 and LN-Cap, and then the PC-3 cells were selectively killed by HVJ-E because the expression level of HVJ-E receptors on PC-3 cells is higher than that on LN-Cap cells. Thus, HVJ-E has been attracting considerable attention as a new type of therapeutic material for cancer therapy. From these perspectives, we develop a novel anti-cancer nanofiber mesh using HVJ-E (Figure 1). The nanofibers are developed by electrospinning poly(*ε*-caprolactone) (PCL). The surface of PCL nanofiber is coated with polyelectrolytes via layer-by-layer (LbL) assembly to construct the precursor film for HVJ-E immobilization. Release of the HVJ-E particles in physiological conditions and inductions of tumor cell deaths are studied through *in vitro* tests.

## 2. Results and Discussion

### 2.1. Preparations of HVJ-E Immobilized PCL Nanofibers via LbL Method 

**Figure 2:**
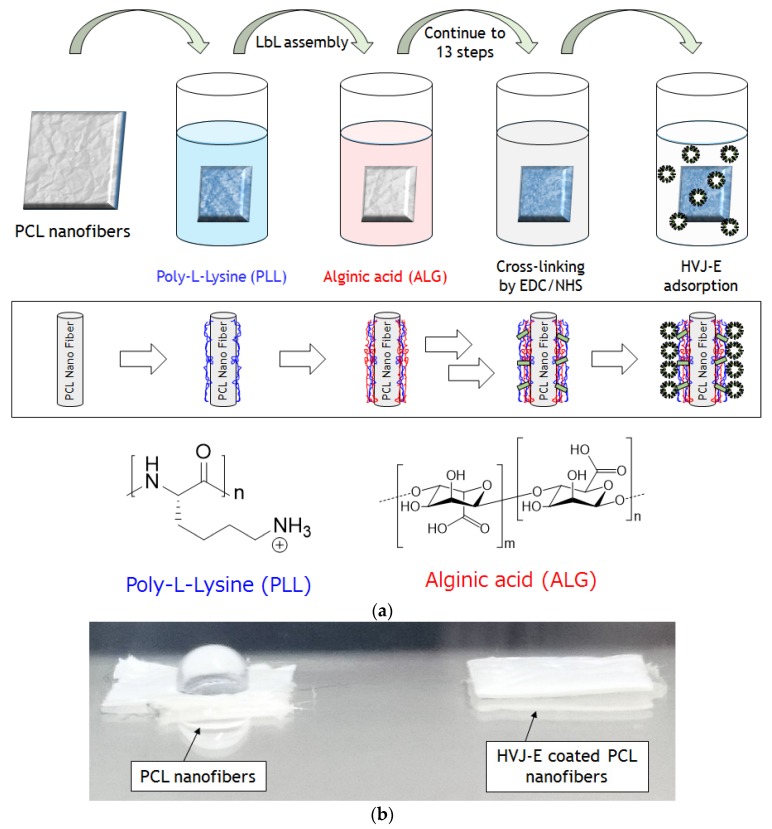
(**a**) Layer-by-Layer coating on PCL nanofibers with poly-L-Lysine (PLL) and alginic acid (ALG) via alternative deposition of polymers. After LbL coating, the fibers were crosslinked by EDC/NHS. HVJ-E was then adsorbed on the PLL outermost surface; (**b**) Photograph of water droplet on the PCL nanofiber mesh with (right) and without (left) HVJ-E immobilization.

We choose to make nanofiber meshes with poly(*ε*-caprolactone) (PCL) in this study because PCL is biocompatible, biodegradable, and widely used in the biomaterial/biomedical fields such as tissue engineering and drug delivery [18,19,20]. Firstly, *ε*-caprolactone monomer was polymerized by a method described previously [21]. The PCL nanofibers were prepared by the electrospinning method. Briefly, PCL was dissolved in 1,1,1,3,3,3-hexafluoro-2-propanol (HFIP) (20 wt/v %) and electrospun for 2 h onto an aluminum substrate using 20 kV at a flow rate of 1 mL/h [22]. To immobilize HVJ-E particles on PCL nanofiber meshes, layer-by-layer (LbL) assembly technique was employed (Figure 2a). The LbL method is widely used because it can be applied on many substrates such as glass, gold, plastic, and fibers [23,24,25,26]. The LbL films have been extensively used in the biological/biomedical fields because the components of the films can be taken from biocompatible precursors such as hyaluronic acid, chitosan, alginic acid (ALG), and poly-L-lysine (PLL). Some researchers have chosen to use LbL as a method to retain protein activity in an immobilized state to deliver the protein to a desired location. Guilot *et al*., for example, reported that the bone morphogenetic protein 2 (BMP-2) immobilized PLL/hyaluronic acid based LbL films on a bone implant enhanced the bone regeneration in an embedded site [27]. The result showed that BMP-2 was delivered to an embedded site without any inactivation of protein activity. We have recently reported the HVJ-E immobilization on LbL films and confirmed the viral protein activity of the HVJ-E [28]. In this study, the PCL nanofibers were immersed into polyethylene amine (PEI) solution to prepare a precursor layer on the fibers. Then the PEI adsorbed fibers were immersed into ALG solutions, followed by washing with HEPES/NaCl buffer. The ALG adsorbed fibers were dipped into PLL solutions to form the polyion complex. In order to make a stable structure in the cell culture media, the LbL layer was crosslinked. Both 1-Ethyl-3-(3-dimethylaminopropyl) carbodiimide (EDC) and *N*-hydroxysuccinimide (NHS) were used to crosslink the carbonic acid groups of ALG and amino groups of PLL in LbL multilayers [29]. The FTIR spectrum was measured to confirm the existence of crosslinked LbL multilayer on PCL nanofibers (Figure S1). The broad weak peak in the spectrum at 3200–3400 cm^−1^ and at 1515–1600 cm^−1^ were assigned to the N-H stretching and amide II in crosslinked PLL-ALG structure in LbL multilayer, respectively. This observation suggested the successful immobilization of LbL multilayer on PCL nanofibers. After the crosslinking reaction, PLL outermost crosslinked nanofibers were immersed into HEPES/NaCl solutions containing 3000 Hemagglutinating unit/mL (HAU/mL) of HVJ-E to obtain HVJ-E adsorbed LbL coated PCL nanofibers. HVJ-E has an anionic zeta potential in physiological pH and could adsorb on the final PLL cationic positively charged layer [30]. As shown in Figure 2b, the nanofiber mesh became hydrophilic after the HVJ-E immobilizations due to the surface proteins located on HVJ-E envelope [31]. 

### 2.2. Characterizations of HVJ-E Coated PCL Nanofibers

#### 2.2.1. SLM and SEM Observations.

**Figure 3:**
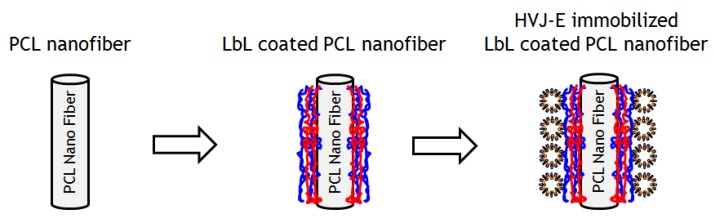

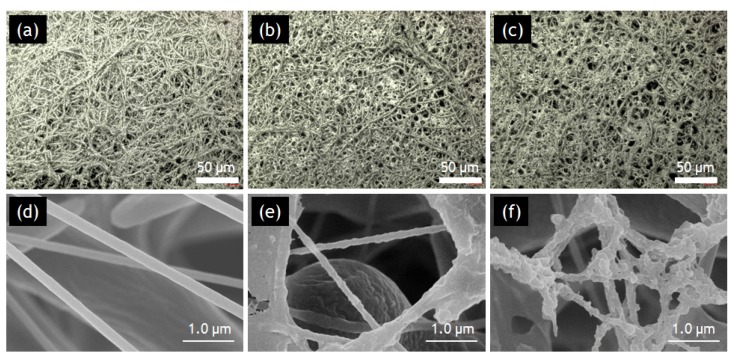
SLM (**a**–**c**) and SEM (**d**–**f**) images of PCL(**a**,**d**), LbL coated (**b**,**e**), and HVJ-E coated PCL nanofibers (**c**,**f**).

Firstly, prepared PCL fibers, LbL coated PCL nanofibers and HVJ-E coated PCL nanofibers were observed by scanning laser microscopy (SLM) and scanning electron microscopy (SEM). SLM images of PCL, LbL coated PCL nanofibers, and HVJ-E immobilized LbL coated PCL nanofibers are shown in Figure 3a–c, respectively. Some membrane-like structures are observed between the adjacent nanofibers after LbL coating, which could be caused by the assembly of multilayer films in the limited space in between the adjacent fibers. In order to observe the immobilized HVJ-E particles on the nanofibers, the magnified images of nanofibers were also observed by SEM and shown in Figure 3d–f. The average diameter of PCL nanofibers was estimated to be 153 ± 42 nm (an average of 28 fibers). Although it was difficult to identify the layered structure and HVJ-E particles, the surface morphology significantly changed after the immobilization of HVJ-E particles [30]. These results clearly indicate that HVJ-E particles were successfully coated onto the surface of PCL nanofibers.

#### 2.2.2. CLM Observations

**Figure 4:**
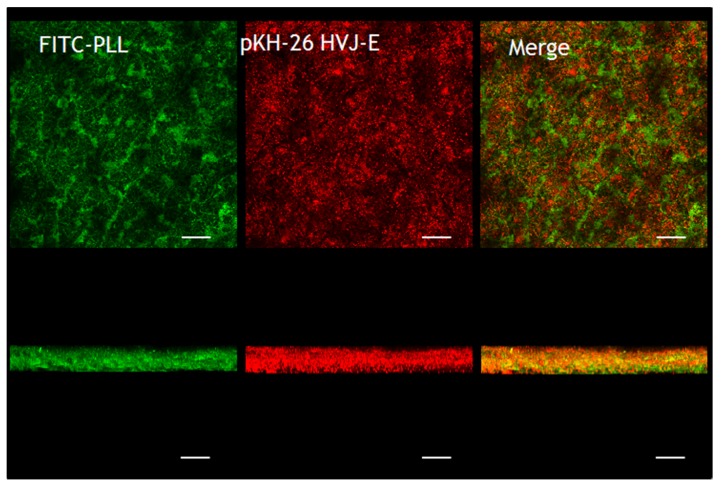
Fluorescent images of HVJ-E immobilized nanofibers observed by CLM. PLL and HVJ-E were stained with FITC (green) and pKH-26 (red), respectively. Top-view (top) and cross-sectional (bottom) images of the nanofiber mesh (scale bars = 50 µm).

One of the great advantages of nanofiber structure compared to two dimensional substrates is the large surface area and porosity of the nanofiber [32]. Therefore, a large amount of molecules can be deposited onto the fiber surface. But it is normally difficult to achieve homogeneous coating within a thick nonwoven nanofiber mesh because the concentration is gradually changed in the thickness direction. Therefore, confocal laser microscopy (CLM) was used to observe the distribution of HVJ-E particles within the nanofiber. Figure 4 shows the confocal microscope images of HVJ-E immobilized nanofibers. PLL and HVJ-E were stained with FITC (green) and pKH-26 (red), respectively. It can be seen that relatively uniform coatings were formed throughout the nanofiber mesh. The bottom images are vertical images for the nanofiber reconstructed by combining multiple sections of CLM images. These images show that both green and red fluorescence were observed not only on the surface of PCL-nanofibers but also inside the fiber structures.

### 2.3. HVJ-E Releasing from the Nanofibers

**Figure 5:**
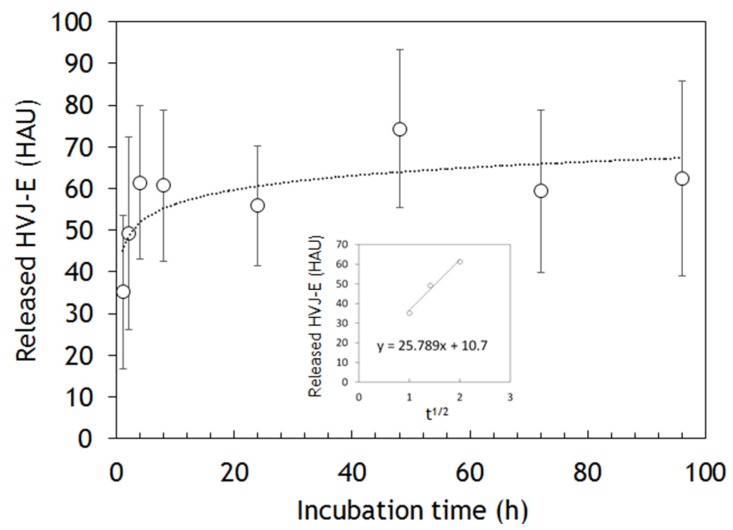
The HVJ-E release kinetics from nanofiber meshes measured by UV-vis. The inserted graph plots the released HVJ-E against the square root of a time.

The release of HVJ-E particles from the nanofibers was examined by measuring the absorbance at 280 nm by UV-vis (Figure 5). The desorption of HVJ-E particles was achieved by using ionic strength and temperature changes to interfere with the electrostatic interactions between HVJ-E and PLL. Therefore, the HVJ-E release experiment was conducted in Dulbecco’s phosphate buffer saline (PBS) at 37 °C. The HVJ-E particles were released over time, and it reached equilibrium at around 60 hemagglutinin unit (HAU) of HVJ-E after 8 h. Figure S2 in the supplementary material shows the effect of the amount of HVJ-E on the cytotoxicity of PC-3 cells. According to this data, 60 HAU of HVJ-E is enough to induce the cell cytotoxicity. The inserted graph plots the released HVJ-E during the first four hours against the square root of a time. Interestingly, it fits to the Higuchi model which describes the release rate of drugs from a matrix system [33]. 

### 2.4. Cytotoxic Assay

**Figure 6:**
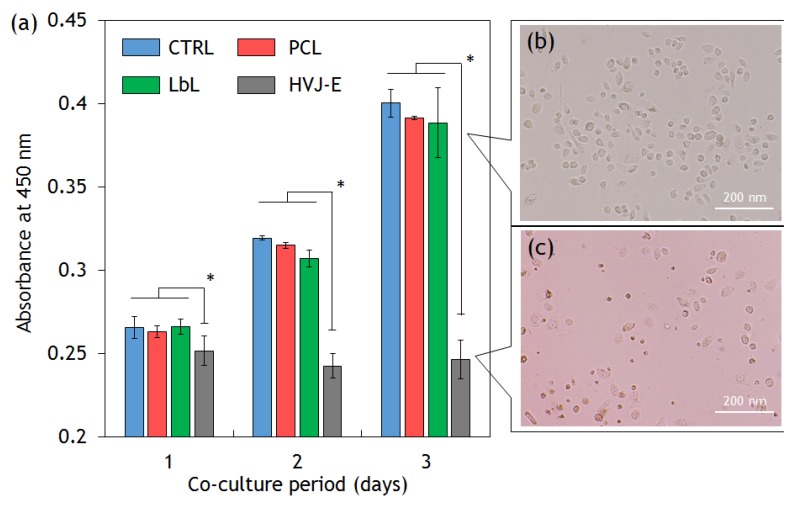
(**a**) Cytotoxic assays. The viability of PC-3 cells was tested using cell counting kit-8 assay. The cells were co-cultured with PCL, LbL coated (PLL outermost), HVJ-E immobilized (HVJ-E) nanofiber mesh (student’s t test *p* < 0.01). The phase contrast images of PC-3 cells (**b**) without HVJ-E immobilized nanofiber mesh; and (**c**) with HVJ-E immobilized nanofiber mesh.

In this part, we attempted to confirm whether the released HVJ-E from nanofibers induce tumor cell death. We conducted *in vitro* tests to observe the viability and proliferation ability of tumor cells. Metastatic prostate cancer PC-3 cells were cultured in 24-well plates. PC-3 cells were chosen because previous study suggested that the PC-3 cells have a HVJ-E receptor GD1a that enables them to capture HVJ-E particles [17]. A 1 cm × 1 cm of nanofiber mesh was added to the cell culture wells for co-cultivation with nanofibers and cells were cultured for 72 h (Figure 6a). Compared with the control (medium only), there were no significant decreases in the proliferation of the cells in the presence of PCL and LbL nanofibers. This result indicates that nanofiber itself shows no cytotoxicity for PC-3 cells. By contrast, the cell proliferation was significantly suppressed in the presence of HVJ-E immobilized nanofiber mesh. This result corresponds well to the *in vitro* HVJ-E release study and cytotoxic assay as shown in Figure 5 and Figure S1, respectively. Figure 6a shows the phase contrast image of PC-3 cells without HVJ-E immobilized nanofiber mesh. The cells kept their circular shape. On the other hand, dead cells were observed when cells were co-cultured with HVJ-E immobilized nanofiber mesh, indicating that released HVJ-E particles caused tumor cell death (Figure 6b). 

Because Matsushima-Miyagi *et al*. reported that the inside RNA fragments of HVJ-E were recognized by retinoic acid-inducible gene I (RIG-I) [34]. Although the molecular mechanisms of apoptosis induction are still under investigation, the paper hypothesized that the mechanism involved is the RNA fragments of HVJ-E induced a signaling process that attacks the cells via the RIG-I/mitochondrial antiviral signaling protein (MAVS). Signaling downstream led to the cells upregulating tumor necrosis factor related apoptosis ligand (TRAIL) expressions. Then the TRAIL could conjugate with tumor necrosis factor receptor (TNF-R) and cause cell apoptosis. TNF-R4 and TNF-R5, the apoptosis inducing receptors, are strongly expressed on tumor cells. Therefore, HVJ-E could selectively induce apoptosis in cancer cells. These mechanisms could occur via released HVJ-E from PCL nanofibers in this experiment.

## 3. Experimental Section 

### 3.1. Materials

*ɛ*-Captolactone monomer was purchased from Tokyo Kasei (Tokyo, Japan). Poly-L-lysine hydrobromide (M_w_ 30,000–70,000), sodium alginate (low viscosity), and pKH-26 staining kit were purchased from Sigma Aldrich (St. Louis, MO, USA). Freeze dried HVJ-E (GE-016) was purchased from Ishihara Sangyo Kaisha, Ltd. (Osaka, Japan). RPMI-1640 and Dulbecco’s phosphate buffer saline (without calcium) were purchased from Nacalai tesque (Kyoto, Japan). HEPES and cell counting kit-8 were purchased by Dojindo (Kumamoto, Japan). Prostate cancer PC-3 cell line was provided by RIKEN Cell Bank (Tsukuba, Japan). 

### 3.2. Fabrication of Nanofibers

PCL was synthesized by ring-opening polymerization with tin octanoate [21]. The obtained PCL was dissolved in 1,1,1,3,3,3-Hexafluoro-2-propanol (HFIP) solution at the concentration of 20 wt/v%. The solution was then electrospun into nanofibers through 23 G syringe-type needle at a constant flow rate of 1.0 mL/h by 20 kV onto an aluminum foil plate. After electrospinning, the fibers were vacuum dried to remove HFIP completely.

### 3.3. Preparation of Crosslinked Layer-by-Layer Assembled Films on PCL Nanofibers

Poly-L-lysine (PLL) and alginic acid (ALG) were dissolved in pH 7.4 of HEPES/NaCl buffer (10 mM HEPES and 150 mM NaCl). The concentration of each solution was adjusted to the concentration of 1.0 mg/mL and 5.0 mg/mL respectively. Firstly, PCL nanofibers were dipped into 0.1 mg/mL of poly ethylene imine (PEI) solution for 10 min to fabricate a precursor film on the fiber. The fibers were then dipped into HEPES/NaCl buffer for 2 min. The PEI coated nanofibers were dipped into the ALG solution for 10 min, followed by washing with HEPES/NaCl buffer for 2 min. The ALG outermost fiber was dipped into PLL solution for 10 min to form the LbL multilayer films. This adsorption/washing process was continued alternatively to assemble the LbL multilayer on PCL nanofibers. The EDC and NHS were dissolved in HEPES/NaCl buffer and the concentration was adjusted to 800 mM and 200 mM. These solutions were mixed together before the crosslinking reaction. The PCL nanofiber with PLL on the outermost fibers was dipped into an EDC-NHS mixed solution for 4 h at 4 °C. After the reaction, the fibers were dipped into HEPES/NaCl buffer overnight to remove unreacted EDC. Attenuated total reflection Fourier transform infrared (ATRFTIR) spectrum was measured on a FTIR spectrophotometer (Perkin-Elmer Spectrum One, Waltham, MA, USA) equipped with universal ATR sampling accessory for surface analysis. The samples had been dried *in vacuo* at room temperature before ATR-FTIR measurement. All of the measurements were performed under identical conditions (number of scans: 24 times, resolution: 8 cm^−1^).

### 3.4. HVJ-E Adsorption on the Crosslinked Layer-by-Layer Film on PCL Nanofibers

The HVJ-E fibers were prepared first by crosslinking PLL outermost LbL coated fibers, followed by sterilization with UV light for 15 min. The crosslinked LbL nanofibers were then dipped into 3000 HAU/mL of HVJ-E solution (HEPES/NaCl buffer) for 2 h. The fibers were then washed two times with HEPES/NaCl buffer.

### 3.5. Characterization of PCL Nanofibers with/without HVJ-E Coating

Scanning Electron Microscope (SEM) image was taken by SU-8000 (Hitachi, Japan) for the examination of nanofiber shapes after platinum coating. Confocal microscope analysis was performed by SP-5 (Leica, Germany). FITC labelled PLL and ALG were assembled on PCL nanofibers as the above described method. Then the sample was observed by Ar laser and He/Ne laser. 

### 3.6. HVJ-E Release from HVJ-E Immobilized LbL Coated Nanofibers

The 1 cm × 1 cm of HVJ-E immobilized LbL coated nanofibers were immersed in 5 mL of PBS. The solution was incubated at 37 °C in the stirring machine (55 times/min). 1 mL of the solution was collected at 1, 2, 4, 8, 24, 48, and 72 h and 1 mL of PBS was added each time, respectively. The absorbance at 280 nm of each sample was measured to examine the concentration of HVJ-E solution and the value was converted to concentration by the prepared standard curve.

### 3.7. Cytotoxic Ability of HVJ-E Coated PCL Nanofibers

The 10,000 cells of PC-3 cells were cultured in a 24-well plate for 24 h. 1cm × 1 cm of PCL fibers, LbL coated nanofibers and HVJ-E fibers were put in each well and again cultured for 24, 48, and 72 h independently. After each time period, the fibers were removed and 50 µL of cell counting kit-8 reagent was then added to each well and incubated for 2 h at 37 °C. The absorbance was measured at 450 nm by an UV plate reader.

## 4. Conclusions

In this study, we fabricated HVJ-E immobilized electrospun nanofiber meshes by using the LbL technique. As the HVJ-E particles are fragile, first we prepared precursor LbL film on the surface of PCL nanofibers with PLL and ALG via electrostatic interaction. Then the HVJ-E particles were immobilized on the cationic PLL outermost surface. These immobilized HVJ-E particles were released from the nanofibers under physiological condition. *In vitro* cytotoxic assays demonstrated that the released HVJ-E from nanofibers induced cancer cell deaths. We believe that the HVJ-E nanofiber offers significant promise for the cancer immunotherapy because the proposed method perhaps regarded as a simple, versatile, and an inexpensive bottom-up nanofabrication technique. In addition, this surface immobilization technique is possible to perform on anti-cancer drug incorporated nanofibers that enables the fibers to show chemotherapy and immunotherapy at the same time to effectively eradicate tumor cells *in vivo*. 

## Supplementary Files

Supplementary File 1
