# Supplementary material for: Inactivated Sendai Virus (HVJ-E) Immobilized Electrospun Nanofiber for Cancer Therapy
Source: Materials (Basel). 2015 Dec 26;9(1):12. doi: 10.3390/ma9010012 (PMC5456544; doi:10.3390/ma9010012)
Supplement: Supplementary File 1 [file materials-09-00012-s001.pdf]

Supplementary Materials: Inactivated Sendai Virus (HVJ-E) Immobilized Electrospun Nanofiber for Cancer Therapy

Takaharu Okada, Eri Niiyama, Koichiro Uto, Takao Aoyagi and Mitsuhiro Ebara

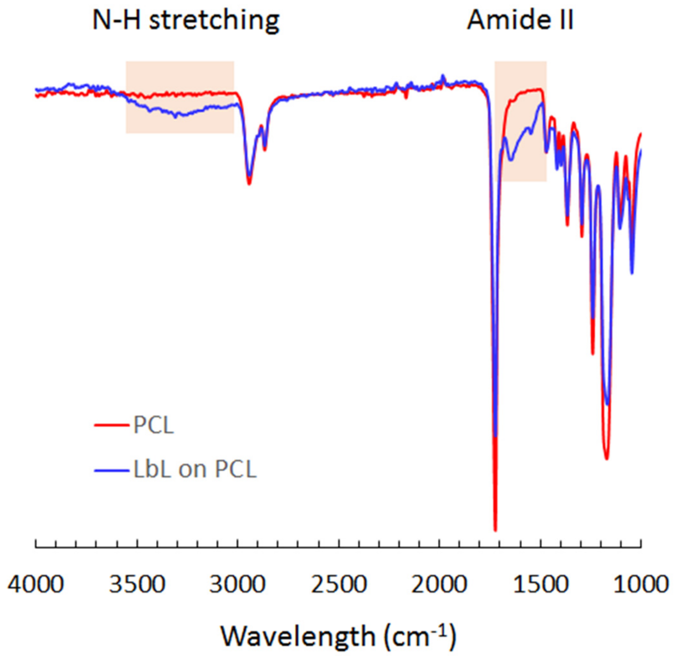

Figure S1. FTIR spectrum of bare PCL nanofibers and LbL coated PCL nanofibers were measured for confirming the existence of LbL multilayer on PCL nanofibers, respectively.

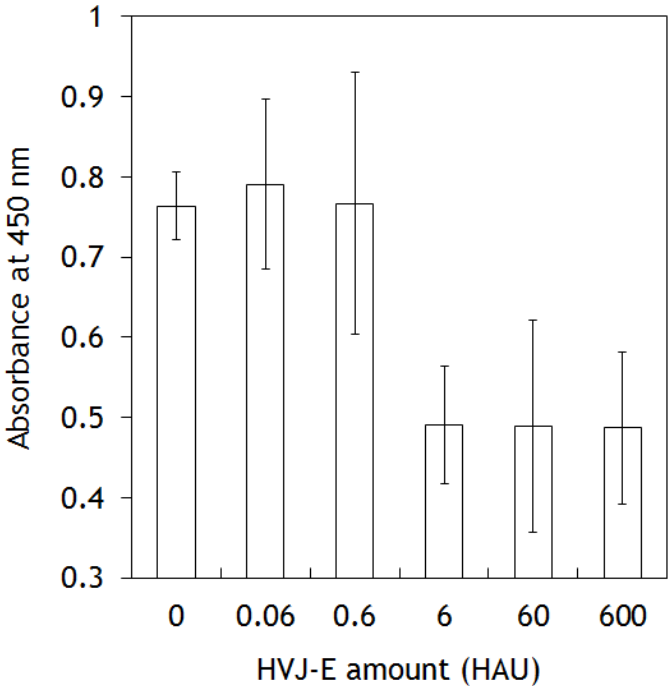

Figure S2. The cell cytotoxicity assays for 10,000 cells of PC-3 cells co-cultured with 0.06–600 HAU of HVJ-E for 24 h, respectively. After the co-culture, the 10 μL of cell counting kit-8 was added to each wells and incubated for 2 h at 37 °C. Then the absorbance at 450 nm of each wells were measured by the UV plate reader.
